# COVID-19 Related Coagulopathy: A Distinct Entity?

**DOI:** 10.3390/jcm9061651

**Published:** 2020-05-31

**Authors:** Benjamin Marchandot, Laurent Sattler, Laurence Jesel, Kensuke Matsushita, Valerie Schini-Kerth, Lelia Grunebaum, Olivier Morel

**Affiliations:** 1 Université de Strasbourg, Pôle d’Activité Médico-Chirurgicale Cardio-Vasculaire, Nouvel Hôpital Civil, Centre Hospitalier Universitaire, 67000 Strasbourg, France; benjaminmarchandot@gmail.com (B.M.); Laurence.JESEL-MOREL@chru-strasbourg.fr (L.J.); matsuken_22@yahoo.co.jp (K.M.); 2 Université de Strasbourg, Pôle de Biologie, Département d’Hémostase, Centre Hospitalier Universitaire, 67000 Strasbourg, France; laurent.sattler@chru-strasbourg.fr (L.S.); lelia.grunebaum@chru-strasbourg.fr (L.G.); 3 UMR INSERM 1260, Regenerative Nanomedicine, Faculté de Pharmacie, Université de Strasbourg, 67400 Illkirch, France; valerie.schini-kerth@unistra.fr

**Keywords:** coronavirus disease 2019, COVID-19, thrombosis, hemostasis, microparticles, endothelium, antiphospholipid antibodies

## Abstract

The coronavirus disease 2019 (COVID-19) pandemic has impacted healthcare communities across the globe on an unprecedented scale. Patients have had diverse clinical outcomes, but those developing COVID-19-related coagulopathy have shown a disproportionately worse outcome. This narrative review summarizes current evidence regarding the epidemiology, clinical features, known and presumed pathophysiology-based models, and treatment guidance regarding COVID-19 coagulopathy.

## 1. Introduction

Since December 2019, the coronavirus disease 2019 (COVID-19) has spread globally, infecting more than 3 million people and causing more than 213,273 deaths in over 200 countries as of 28 April 2020 [1,2,3]. Whereas COVID-19 is primarily a respiratory infection caused by severe acute respiratory syndrome coronavirus 2 (SARS-CoV-2), it has important systemic effects on the cardiovascular and immune systems [4,5]. COVID-19 patients present with a variety of phenotypes that range from asymptomatic to profound rapid multiple-organ dysfunction syndrome and death. Early reports of critically ill patients demonstrated that the development of coagulopathy was one of the most significant poor features [6,7], leading to a prompt response from both national and international societies of thrombosis and hemostasis to guide recognition and management of COVID-19-related coagulopathy [8,9,10,11,12,13,14]. A variety of potential risk factors for thrombosis have been proposed, including severe hypoxemia, inflammation, and endothelial dysfunction, on top of well-known features such as immobilization, respiratory failure, mechanical ventilation, and central venous catheter use: Hallmarks of critically ill patients.

This narrative review summarizes current evidence regarding the epidemiology, clinical features, known and presumed pathophysiology-based models, and treatment guidance regarding COVID-19 coagulopathy.

## 2. Pathogenesis of COVID-19

SARS-CoV-2 was initially identified in Wuhan, China in December 2019 [15]. A member of the Coronaviridae family, SARS-CoV-2 is an enveloped virus with non-segmented, single-stranded, positive-sense RNA genome [16,17]. SARS-CoV-2 binds its viral spike (S) proteins to angiotensin-converting enzyme 2 (ACE2) proteins for cell entry and uses the cellular serine protease transmembrane protease serine 2 (TMPRSS2) for S protein priming [17,18,19,20].

ACE2 is highly expressed in lung alveolar cells (principally type II alveolar cells) and serves a role in lung protection, and therefore viral binding to this receptor deregulates a lung protective pathway, contributing to viral pathogenicity [21,22]. Other significant locations comprise endothelial cells of blood vessels and the heart, pericytes, adipocytes, and neural cells [23]. The virus is thought to spread mainly from human to human through respiratory droplets produced by infected individuals [24]. Rapid spread has been related to (i) a similar detected viral load in symptomatic and asymptomatic patients with COVID-19, (ii) a high transmission as a large number of patients carry no or mild symptoms, and (iii) a basic reproduction number (R0) estimated to be 2.2 [25,26]. Initial symptoms of COVID-19 are non-specific and include fever, cough, shortness of breath, headaches, myalgias, diarrhea, and fatigue [15,16]. Large case series from China including 72,314 patients with COVID-19 have indicated that the clinical severity was mild (no or mild pneumonia) in 81%, severe (dyspnea, respiratory frequency ≥30/min, blood oxygen saturation ≤93%, partial pressure of arterial oxygen to fraction of inspired oxygen ratio <300, and/or lung infiltrates >50% within 24 to 48 h) in 14%, and critical (respiratory failure, septic shock, and/or multiple organ dysfunction or failure) in 5% of the population [27].

### 2.1. Hemostasis Parameters in COVID-19

The first hint of a noxious impact and frequent feature of hemostasis disorders in patients with novel coronavirus pneumonia was reported by Guan et al. on 28 February 2020 [6]. In this initial cohort of 1099 hospitalized patients with COVID-19, 46.4% presented with an increased D-Dimer level above 0.5 mg/L. A higher D-Dimer level was evidenced among severe compared to non-severe COVID-19 patients (59.6% vs. 43.2%, respectively) as for the occurrence of a primary composite end-point including intensive care unit (ICU) admission, invasive mechanical ventilation requirement, and death. Later reports of critically ill patients with abnormal coagulation parameters confirmed poor prognostic features [7,28,29]. In a cohort of 191 hospitalized patients with COVID-19, non-survivors had D-Dimer levels on admission greater than 1 mg/L in 81% (81% vs. 24%, *p* < 0.0001) [28]. Additionally, this threshold predicted an 18-fold increase in the odds of dying. Of note in this report, severe thrombocytopenia < 100 × 109/L only occurred in 20% of non-survivors, similarly to prolonged prothrombin time (PT) ≥ 16 s in 13%. Finally, a third study from China by Tang et al. [7] confirmed higher D-Dimer levels (2.12 vs. 0.61 μg/mL, *p* < 0.001) and a mild increase in PT (15.5 vs. 13.6 s, *p* < 0.001) in non-surviving patients. In this study, early increased fibrinogen and slightly decreased antithrombin levels did not reach statistical significance between survivors and non-survivors. Following early reports, the body of evidence now suggests that moderate and severe COVID-19 patients are likely to present prolonged PT, activated partial thromboplastin time (aPTT), and elevated D-Dimer levels with subsequent poorer outcomes [30,31,32,33].

### 2.2. Disseminated Intravascular Coagulation in COVID-19

Tang et al. reported that 71.4% of non-survivors and 0.6% of survivors showed evidence of disseminated intravascular coagulation (DIC), suggestive of a frequent manifestation with severe COVID-19 [7]. The pathophysiology of DIC in the setting of sepsis and acute respiratory distress syndrome (ARDS) is multifactorial and involves a complex interplay between cellular and plasmatic elements of the hemostatic system with immune-mediated exhaustion of the coagulation and fibrinolytic systems promoting bleeding and thrombosis in the same patient [34,35,36]. Severe infections and sepsis are a leading cause of DIC, and the pro-inflammatory and immune activation observed in severe COVID-19 is likely sufficient to trigger DIC. Such involvement of the hemostatic system in severe novel coronavirus pneumonia surprised the intensive care and hemostasis community due to the high likelihood to develop DIC [37]. Indeed, the frequency of DIC in COVID-19 patients is much higher than that reported for severe SARS with a total of 2.5% of SARS patients showing evidence of DIC [38]. As the COVID-19 pandemic keeps on progressing, additional studies are warranted to investigate the incidence of DIC and address the propensity of SARS-CoV-2 to engage both innate immune and hemostatic systems.

### 2.3. D-Dimer Generation in COVID-19: Different Pathways?

Physicians at the front line in the fight against the COVID-19 disease reported early concerns about markedly elevated D-Dimer levels: Are they any different from previous studies or similar conditions? Comparing the coagulation parameters between severe pneumonia induced by SARS-CoV-2 and non-SARS-CoV-2, Yin et al. reported in a retrospective analysis a higher platelet count (215 ± 100 vs. 188 ± 98, *p* < 0.015) but no differences regarding other coagulation parameters including PT and D-Dimer [39]. This analysis was restricted to severe novel coronavirus pneumonia patients admitted to the intensive care unit, and unpublished data still indicate unusual higher levels of D-Dimers even in mild to moderate COVID-19 patients.

Hemostatic changes and high D-Dimer levels in COVID-19 patients have been explained by (i) excess thrombin generation and early fibrinolysis shutdown secondary to endothelial activation induced by the infectious trigger [40,41,42], (ii) severe hypoxemia known to stimulate thrombosis through both increased blood viscosity and hypoxia-inducible transcription factors [43], and finally (iii) local pulmonary thrombotic phenomena with a high frequency of pulmonary micro-thrombosis in small autopsy series [44,45]. Such focal thrombotic lung injury paved the way for the concept of a focal pulmonary thrombosis phenomenon in 2019 novel coronavirus pneumonia [46].

### 2.4. Gaps in Evidence

Since the initial description of COVID-19 abnormal coagulation parameters associated with poor prognosis, COVID-19 has been associated with noteworthy hemostatic features. First thought to mimic DIC, reports showed less prominent thrombocytopenia and consumption of coagulation proteins. No data have yet focused on thrombotic microangiopathy, and new research is warranted to assess a potential interaction between COVID-19, Von Willebrand, and ADAMTS-13. As most papers have concentrated mainly on thrombin generation to account for the hypercoagulability of COVID-19, SARS-CoV-2-induced platelet hyper-reactivity deserves deeper attention [47]. In addition to thrombosis and hemostasis, emerging evidence indeed supports a putative role of platelets in host defence against infections [48]. As the angiotensin-converting enzyme (ACE2) is also expressed in hepatic tissues, viral binding to a hepatic receptor may trigger liver dysfunction [49]. Finally, the role for antiphospholipid antibodies (see below) and immune thrombocytopenia in COVID-19-related thrombosis requires further investigation [50,51].

## 3. Pathophysiology of COVID-19-Induced Coagulopathy 

Higher thrombotic burden in the acute phase of COVID-19 relies on a complex interplay between pro-inflammatory cytokine/chemokine release, increased endothelial dysfunction/damage, and potential sepsis-induced coagulopathy development in severe cases, all promoting coagulation activation. The surprising highly pro-thrombotic features of COVID-19 seem to hail from (i) severe and prolonged hypoxemia known to stimulate thrombosis, (ii) high incidence of cytokine storms in critically ill patients, and finally (iii) a putative role of local pulmonary thrombotic phenomena. 

### 3.1. Inflammation

Inflammation has been accepted as a common pathway through which various risk factors trigger thrombogenesis [52,53]. Cytokines and chemokines have been associated with an important role in immunity and immunopathology during viral infections [54,55,56]. The immune response to acute SARS-CoV-2 infection and the accompanying surge of cytokines and inflammatory mediators (interleukin (IL)-6, IL-7, IL-22, C-x-C motif chemokine 10, etc.) can lead to activating pro-coagulant pathways. Inflammatory cytokines, together with endothelial injury, can up-regulate tissue factor expression and further drive a pro-thrombotic state [37,57]. In coronary arteries, circulating cytokines can stimulate macrophages within the plaque to increase local cytokine production and provoke tissue factor overexpression that renders lesions more thrombogenic. This mechanism of a local, intraplaque response to systemic stimuli was referred to as an “echo” phenomenon by Libby [58]. Systemic cytokines can stimulate leukocyte adhesion molecule expression on the endothelial cells overlying established atheroma, boosting local recruitment of these inflammatory cells [58].

Early reports from China described severely elevated levels of inflammatory biomarkers and cytokines such as IL-6, IL-1β, C-reactive protein, Tumor necrosis factor (TNF)-alpha, granulocyte-colony stimulating factor, and ferritin [15,28,59,60]. Advanced stages of COVID-19 have been linked to cytokine storm syndromes and identified patients at higher risk of progressing to severe diseases including DIC, acute myocardial injury due to myocarditis or stress-cardiomyopathy, and death [61,62,63]. Siddiqi et al. have proposed a schema to assess the severity of “systemic hyperinflammation” in COVID-19 [64].

According to this paradigm, the host response to COVID-19 is first localized in the lung parenchyma, but a systemic surge in pro-inflammatory cytokines can occur. Defined as a “cytokine storm,” the incidence, role, and specific involvement of cytokine storms is yet to be addressed in SARS-CoV-2, and its interactions with hemostatic changes as observed in previous viral diseases [65,66,67] remain to be established.

### 3.2. Endothelial Activation 

Clinical and pre-clinical evidence supports the hypothesis that the endothelium is a key target organ of COVID-19 [68,69]. The importance of the endothelium in the pathogenesis of COVID-19 infection was recently emphasized by data demonstrating direct endothelial cell infection and endotheliitis in the time course of SARS-CoV-2 infection [70]. In humans, the angiotensin-converting enzyme 2 (ACE2) receptor has been found in the lung epithelium (in particular the type II pneumocyte) and the myocardium, and is highly expressed in arterial and venous endothelial cells [71]. Endothelial cell activation/damage due to the virus binding to the ACE2 receptor promoting acute inflammation and hypercoagulation may be of paramount importance to explain the high thrombotic burden observed.

Under physiological conditions, pulsatile shear stress increased ACE2 expression, promoting nitric oxide production, and reducing inflammation and proliferation in vascular endothelial cells [72]. The recent description of higher ACE2 expression as determined by RNA sequencing and confirmed by proteomic profiling in heart muscle disease patients might explain why heart failure patients could be more vulnerable to heart infection by SARS-CoV-2 [23]. Pericytes, which spread outside the endothelial cell of capillaries and parts of venules, were also suggested to be a key target in SARS-CoV-2 infection and might explain the importance of capillary endothelial cell dysfunction and microcirculation disorder in that setting [23]. General mechanisms of endothelial activation following a cytokine burst include calcium mobilization, oxidative stress, down-regulation of the endothelial nitric oxide synthase-derived nitric oxide formation, plasma membrane remodeling, exposure of procoagulant phospholipid such as phosphatidylserine, procoagulant microparticles (MPs) shedding, tissue factor expression, disruption of the natural anticoagulant shield represented by annexin 5, expression of selectins and cytoadhesins (vascular cell adhesion molecule 1, intercellular adhesion molecule 1), and cytokines release (monocyte chemoattractant protein-1). Within pulmonary microvascular endothelial cells, exposure to various inflammatory stimulus (TNF-alpha, lipopolysaccharide) induced the release of endothelial-derived MPs (EMPs), harbouring ACE and a simultaneous decrease of endothelial cell-surface ACE expression [73]. In a mouse model of lung injury, ACE^+^-EMPs/EMPs were evidenced to be a marker of wet-to-dry lung injury as a possible witness of alveolar capillary barrier alterations. In sepsis, the importance of these findings was emphasized by the demonstration of elevated levels of ACE^+^-EMPs and ACE^+^-EMPs/EMPs in the blood of patients who developed ARDS [73].

Finally, endothelial cell activation/damage due to the virus binding to the ACE2 receptor promoting acute inflammatory and hypercoagulable may be of paramount importance to explain the high thrombotic burden observed [70].

### 3.3. Severe Hypoxemia

Gathering experience from Chinese and Italian physicians at the front line in early phases of the COVID-19 pandemic, much attention has been paid to hypoxemia. Indeed, “silent hypoxemia” has been described in many COVID-19 patients. Patients were hypoxemic as they may have had an oxygen saturation of about 80% room air, but clinically looked comfortable, and not dyspneic or tachypneic. Complementary reports reinforce the high frequency and noxious impact of severe hypoxemia [74,75,76]. ARDS occurred in approximately 40% of 201 patients with 2019 novel coronavirus pneumonia [29]. The COVID-19 pneumonia has lately been addressed as a specific disease with peculiar phenotypes. Its main characteristic is the dissociation between the severity of the hypoxemia and the maintenance of relatively good respiratory mechanics [77]. 

Severe lung inflammation and impaired pulmonary gas exchange in COVID-19 can stimulate thrombosis through a hypoxia-inducible transcription factor-dependent signaling pathway [43]. Furthermore, hypoxia, and ARDS in particular, is known to promote hypoxic pulmonary vasoconstriction and pulmonary hypertension, increase right ventricular afterload, and favor blood viscosity.

### 3.4. Pulmonary Microvascular Thrombosis 

Focal microvascular thrombosis and pulmonary emboli in a small autopsy series have recently been reported [44,45]. Such focal thrombotic lung injury paved the way for the concept of a focal pulmonary thrombosis phenomenon in 2019 novel coronavirus pneumonia [46]. The entry receptor utilized by SARS-CoV is ACE2, which is highly expressed in lung alveolar cells, principally type II alveolar cells. High pulmonary viral loads in the alveoli of COVID-19-infected patients have been reported [78]. The primary infection initiates alveolar injury, resulting in a local inflammatory response, and these microvascular thrombi have been described in an environment of marked inflammatory changes including mononuclear cell infiltrates, virally infected cells, and diffuse alveolar damage [79]. The key question relies upon whether ARDS and/or DIC lead to pulmonary microvascular thrombi on one hand, and, on the other hand, whether focal pulmonary microthrombi can lead to further hypoxemic respiratory failure, enhanced thrombo-inflammation, and hypercoagulability with ARDS and DIC as final consequences: Both options are likely. It may be hypothesized that lung injury results in microthrombus formation further enhanced in the setting of a hypercoagulable state like DIC. 

In this paradigm, the shedding of ACE^+^-MPs by lung microvascular endothelial cells could be of paramount importance [73].

Marongiu et al. developed the view of a “pulmonary thrombosis in 2019 novel coronavirus pneumonia” [46]. The authors emphasized the view of a prothrombotic pulmonary endothelial dysfunction, causing a severe acute inflammation via complement and cytokine release, and a blood coagulation activation with vascular microthrombosis that further induces a local consumption coagulopathy, i.e., a DIC, resulting in ARDS. In light of the Marongiu suggestion that anticoagulant treatment may be helpful by limiting the vicious circle of inflammation-blood coagulation activation-inflammation, thus improving the severely impaired gas exchange in these patients, Rannuci et al. [80] recently showed that the use of an increased dose of low-molecular-weight heparin (LMWH) appeared to reduce the downstream thrombotic effects of the marked inflammatory response to COVID-19 as it seems to decrease the contribution of microvascular thrombosis in severely hypoxemic COVID patients.

## 4. Coagulopathy Disorders Disease in COVID-19 Patients

In line with previous insights from the SARS and A-H1N1 experiences [81,82], data from 1026 patients with COVID-19 in the Chinese population assumed that 40% of patients at the time of hospital admission were considered at high venous thromboembolic (VTE) risk on the basis of a Padua Prediction Score ≥ 4 [83].

### Prevalence of Venous Thromboembolic Disease in COVID-19 

While a number of studies have shown that coagulation dysfunction is preponderant in patients with severe novel coronavirus pneumonia, only a few studies in the available literature have focused on the prevalence of VTE in COVID-19 patients. Early case reports [84,85,86] reporting deep vein thrombosis and/or acute pulmonary embolism (APE) paved the way for a more comprehensive insights into VTE events in larger cohorts. The first retrospective registry cohort of 25 APE-suspected COVID-19 patients in China with computed tomography pulmonary angiography showed that those with confirmed APE (*n* = 10) had D-Dimer levels higher than 7000 ng/mL [87]. In 91 hospitalized patients with severe COVID-19, VTE patients accounted for 25%, were older, and showed increased coagulopathy abnormalities and thrombotic susceptibility (lower lymphocytes count, longer aPTT, and higher D-Dimer levels). In this study proposed by Ciu et al. [88], a D-Dimer cut-off value ≥ 1.5 μg/mL was associated with a sensitivity, specificity, positive predictive value, and negative predictive value of 85.0%, 88.5%, 70.8%, and 94.7%, respectively. Moreover, Klok et al. reported a 31% incidence of thrombotic complications despite systematic thrombosis prophylaxis and no DIC development among a cohort of 184 ICU patients [89]. Similarly, age and coagulopathy defined as spontaneous aPTT time > 5 s (HR 4.1, 95%CI 1.9–9.1) were independent predictors of thrombotic complications.

Finally, Helms et al. lately reported an incidence of 42.6% of thrombotic complications, mainly APE (16.7%) in 150 COVID-19 patients admitted in ICU for hypoxemic acute respiratory failure [90]. Twenty-eight out of twenty-nine patients (96.6%) receiving continuous renal replacement therapy experienced circuit clotting, and three thrombotic occlusions of centrifugal pump occurred in 12 patients supported by extracorporeal membrane oxygenation. Most patients (>95%) had elevated D-dimer levels and fibrinogen, while no patient developed disseminated intravascular coagulation. Most importantly, despite anticoagulation, patients with ARDS secondary to COVID-19 developed significantly more thrombotic complications compared to non-COVID-19 ARDS patients (11.7% vs. 2.1%, *p* < 0.008). The authors proposed in their discussion appealing mechanisms of coagulopathy and pathogenesis of thrombosis in severe hypoxemic COVID-19 patients. They underlined the paramount importance of (i) obvious endothelial inflammation with very high levels of Von Willebrand factor antigen and factor VIII, (ii) hypotheses regarding profound hypoxemia in the pulmonary capillaries that may result in vasoconstriction reducing blood flow and promoting vascular occlusion, and finally (iii) the intriguing high frequency of positive lupus anticoagulant that was detected in 50 patients out of the 57 tested (87.7%). 

Altogether, the increased presence of VTE holds true for COVID-19 patients, most notably among those with severe disease. Currently, the mechanism of these associations remains to be fully characterized.

## 5. Antithrombotic Therapy and COVID-19-Related Coagulopathy

### 5.1. Current Recommendations

Following initiatives from national scientific academies [8,9,10,11,12], the International Society of Thrombosis and Haemostasis (ISTH) promptly proposed an interim guidance on recognition and management of coagulopathy in COVID-19 on 25 March, 2020 [13]. 

In line with coagulation markers at admission and follow up, the provided statement reinforced the need for a risk stratification for all COVID-19 patients including D-Dimers, PT, and platelet count, as these biological parameters are likely to help in stratifying patients who may need admission and close monitoring. As recommended by the previous ISTH guidance [91], regular monitoring of hemostatic markers in critical care units is likely to include fibrinogen on top of platelet count, PT, and D-Dimers as it may detect early DIC development, determine prognosis, and guide critical care support.

Regarding antithrombotic options, the ISTH consensus statement recommended prophylactic dose low-molecular weight heparin (LMWH) in all patients (including non-critically ill) who required hospital admission for COVID-19 infection, in the absence of any contraindications (active bleeding and/or platelet count less than 25 × 109/L) [13].

### 5.2. Heparin and COVID-19

Heparin therefore represents the optimal thrombo-prophylactic and antithrombotic regimen endorsed by contemporary guidelines for patients hospitalized with COVID-19 related illnesses [13]. Tang et al. described that anticoagulant therapy mainly with LMWH appeared to be associated with a better prognosis in 449 severe COVID-19 patients meeting sepsis induced coagulopathy (SIC) criteria ≥4 or with markedly elevated D-Dimer levels (greater than six-fold at the upper limit of normal) [92]. In this paper, however, only 99 patients (22%) had received prophylactic heparin. Heparin therapy has several advantages: (i) It represents, in the time of a pandemic, an easily available anticoagulant therapy, given the initial concerns regarding drug shortages [93]; (ii) incremental anti-inflammatory effects have been reported [94] and it may mitigate cytokine storms in severe COVID-19 patients [95]; (iii) experimental models reported a potential antiviral role of heparin [96,97] still to be confirmed in clinical practice and in the setting of SARS-CoV-2 infection; and finally (iv) there is currently no evidence from randomized clinical trials that any potential therapy improves outcomes in patients with either suspected or confirmed COVID-19 [98]. Given the high prevalence of lupus anticoagulant and changes of standard hemostasis parameters in this particular pathology, monitoring of heparin should not rely on aPTT, but solely on anti-Xa activity.

In summary, a substantial body of evidence suggests how heparin can be beneficial in selected high-risk COVID-19 patients. Conversely, much less is known regarding ambulatory COVID-19 patients’ management and extended thromboprophylaxis after hospitalization.

### 5.3. Gaps in Evidence

Since the clinical experience of managing COVID-19 patients is still limited, gaps remain in the evidence for the management of COVID-19-related coagulopathy. Real incidence of VTE remains unknown in the sparse literature and is probably underestimated because of asymptomatic presentation and/or the lack of systematic imaging. Indeed, routine imaging according to a defined D-Dimer threshold has been proposed in some medical facilities such as ours (arbitrarily greater than ten-fold at the upper limit of normal). Similarly, higher than prophylactic doses of LMWH in those with either (i) extremely high D-Dimer levels at admission, (ii) higher levels of ventilation, or (iii) presenting with ARDS, have been proposed [99]. Validated factors, such as a lack of mobility, advanced age, cancer, previous venous thromboembolism, and elevated D-Dimer levels (> 2 upper the limit of normal range) have been previously associated in the identification of patients who are at risk for symptomatic venous thromboembolism [100]. In the light of a proinflammatory and procoagulant state in COVID-19 patients, extending the research based on previous prolonged thromboprophylaxis experiences in acute medically ill patients may be of particular interest [101,102,103,104].

As the coronavirus outbreak overwhelmed healthcare capacities in some regions, most patients presenting with mild-to-moderate forms have been managed with “home care”, despite ISTH recommendations for hospitalization of patients presenting with three- to four-fold increase of D-Dimers [13]. Therefore, thromboprophylaxis guidance for ambulatory COVID-19 patients is urgently needed. Mirroring prior trials such as the MARINER initiative [104], studies testing either extended thromboprophylaxis after hospitalization or ambulatory thromboprophylaxis in selected COVID-19 patients with direct oral anticoagulants may be of particular interest. 

Finally, in vitro studies aimed to define anti-inflammatory properties and viral inhibition of heparin in the field of COVID-19 represent an appealing subject for future research. Extended reports from tissue plasminogen activator treatment is required [105]. Bleeding seems either rare or under-reported in the setting of COVID-19 and deserves further insights. Finally, the current published COVID-19 experience does not detail the incidence of arterial thrombosis events (stroke, acute coronary syndromes, etc.).

## 6. Future Research 

### 6.1. A Role for Obesity

A high proportion of older patients in a predominantly male population with cardiovascular disease and cardiovascular risk factors have been considered the main determinant of the increased morbidity and mortality [4]. Obesity has recently been increasingly recognized as a major risk factor for severe COVID-19 disease [106,107,108]. Of note, it has long been recognized as a major risk factor for hospitalization and mechanical ventilation [109]. Adipose tissue, besides its role in energy storage, is a potent source of hormones, peptides, procoagulant MPs [110], and cytokines, known as adipokines, and was recently described as an important reservoir of ACE2 [23]. Adipose tissue is involved in the regulation of inflammation (e.g., TNF-alpha, IL-6, macrophage chemo attractant protein-1) and thrombosis (e.g., plasminogen activator inhibitor-1) [111,112]. Since adipose tissue is known to induce an inflammatory state, the potential link between obesity and COVID-19 coagulopathy deserves further insights. Indeed, metabolic syndrome on a broader scale is a hallmark of a pre-existing inflammatory state that may be a necessary condition and/or amplified by COVID-19 in the development of a pro-thrombotic state. The intimated interplay between obesity and COVID-19 severity deserves further insights.

### 6.2. Possible Role of Microparticles in the Development of COVID-19-Induced Coagulopathy

**Figure 1:**
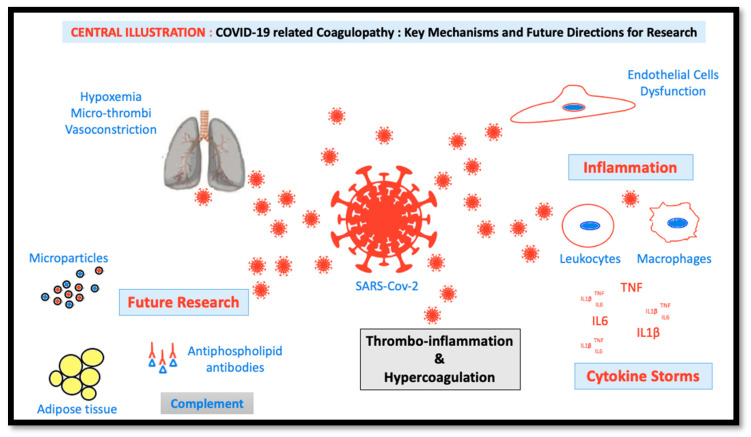
Conceptual figure highlighting major key mechanisms for COVID-19 coagulopathy and future directions in research.

In the time course of COVID-19 infection, it is likely that the cytokine storm promotes a strong activation of circulating blood cells including platelets and leukocytes, and also of endothelial cells lining the luminal surface of the vasculature (Figure 1). This activation process leads to cell blebbing with the shedding of microparticles (MPs) into the circulation. These MPs harbor a mixture of membrane proteins similar to those of the cell membrane of origin, and carry proteins and miRNA to transmit the activator signal to distant cells following cell–cell interaction, thereby contributing to the propagation of the disease. The target cell activation and the circulating MPs promote pro-coagulant responses due to the exposure of tissue factor, and the physiological activator of the coagulation cascade and of negatively-charged phospholipids such as phosphatidyl serine, required for the establishment of the tenase and prothrombinase coagulation complexes ultimately leading to thrombin generation. In the context of cardiovascular disease patients, a vulnerable population in the time course of COVID-19 infection, elevated levels of procoagulant MPs were described in a variety of settings including arterial hypertension, diabetes mellitus, dyslipidemia, obesity, pulmonary embolism, arterial hypertension, acute coronary syndromes, heart failure, etc. [113]. In a large community-based Framingham Heart study, circulating endothelial MP levels were associated with the presence of cardiometabolic risk factors including hypertension and metabolic factors [114], known predisposing factors to severe COVID-19 infections. Recent investigations by our group have indicated that circulating MPs of patients with acute coronary syndrome (ACS-MPs), predominantly of activated endothelial cell and platelet origin, promote pro-oxidant, prothrombotic, and pro-inflammatory responses in endothelial cells leading to endothelial senescence and dysfunction [115]. The stimulatory effect of MPs involves the up-regulation of ACE, which in turn promotes a pro-oxidant response in endothelial cells. Moreover, the ACS-MPs were shown to carry ACE activity, suggesting that they originate predominantly from activated endothelial cells. Another report performed in microvascular lung endothelial cells and in ARDS patients has emphasized the importance of ACE^+^- endothelial-derived MPs as a possible marker of disease severity [73]. Although the presence of ACE2 was not specifically characterized in those studies, these data suggest that circulating MPs are key modulators of various ACE activities within the vascular compartment and could therefore act as an important mediator of COVID-19 pathogenicity. In the context of HIV infection, the transfer of the chemokine receptor between cells by membrane-derived MPs was also demonstrated to be a potent mechanism for cellular human immunodeficient virus 1 infection [116]. In line with this paradigm, recent data have highlighted that exosome, a subtype of extracellular microvesicles shed by endothelial progenitor cells, promotes survival and function of endothelial cells through ACE2 delivery [117]. Preliminary results in COVID-19 patients requiring hospitalization relate a 2-fold generation of procoagulant MPs with respect to values measured in non-COVID-19 patients.

### 6.3. Antiphospholipid Antibodies in COVID-19 Patients

The importance of antiphospholipid (aPLs) antibodies in the development of COVID-19 coagulopathy was stressed by the recent description of multi-cerebral infarctions in 3 patients with antiphospholipid antibodies that were characterized as anticardiolipin IgA, anti B2 glycoprotein I IgA, and IgG [50]. Probable mechanisms of thrombosis in antiphospholipid syndrome comprise endothelial cell dysfunction, platelet activation, complement system activation, inflammatory cell-mediated mechanisms, alteration of anticoagulant properties (tissue factor pathway inhibition, inhibition of the protein C pathway, interference with the action of antithrombin), and reduced fibrinolysis (elevated PAI-1 levels, inhibition of plasminogen binding, and plasma activity) [118]. In our experience, among 150 patients hospitalized for COVID-19 in ICU, the presence of transient lupus anticoagulant (aPL having the ability to prolong clotting time in vitro) was detected in a vast majority of the cases (87.7%) [90]. An inverse correlation between procoagulant MPs levels and aPL activity could be established, suggesting that parts of aPLs could be trapped by circulating MPs, therefore escaping to biological detection. The most important antigen for aPLs is b2-glycoprotein-I (b2GPI), a scavenger molecule, which has a specific binding site for the negatively-charged phospholipid phosphatidylserine (PS). Under physiological conditions, the binding to PS, b2-GPI facilitates clearance of particles and apoptotic bodies from the circulation enabling the maintenance of vascular homeostasis [119]. b2GPI in plasma is normally present as a circular protein; in the case of endothelial cell activation and subsequent remodeling, the exposure of PS at the outer leaflet of the plasma membrane induces conformational changes of b2GPI that “opens up” to a J shape, thus exposing neo epitopes and antibody-binding sites [119]. Membrane remodeling could also contribute to the alteration of natural anticoagulant shield represented by Annexin V, a protein with high PS affinity. Other reports by Nomura and Mobarrez have demonstrated that b2GPI binds preferentially to procoagulant PS-expressing MPs [120], rather than non-PS vesicles. PS-binding antibodies can form structures similar to immune complexes, which are likely to be cleared by Fc receptors through antibody-mediated phagocytosis, reinforcing systemic inflammation and complement activation.

### 6.4. Complement Activation in COVID-19 Patients

Other COVID insights have suggested that thrombotic microangiopathy could include pathogenic complement activation. Antibody-antigens complexes could trigger the classical pathway, inducing the production of C3a and C5a inflammatory markers. Data from murine models have suggested that in the case of the C3 defect, COVID-19 infection was decreased, as witnessed by the decrease of respiratory dysfunction and cytokines levels, despite equal virus loads [121]. Along this line, complement inhibition was suggested to be a promising treatment for severe COVID-19 by reducing the innate immune-mediated consequences of severe SARS-CoV-2 infections [122]. 

## 7. Conclusions

The relationship between COVID-19 and thrombogenesis seems deeply intricate, complex, and still little understood. Concerns and controversies have been raised towards a potential distinct “COVID-19-induced coagulopathy pattern”, while softened statements share the view that it is only part of the well-known “sepsis-induced coagulopathy” or “disseminated intravascular coagulopathy”. More research is needed to explore the interplay between the pulmonary and endothelial localization of ACE2 receptors, hypoxia, and pulmonary microthrombi. There are key elements to investigate in the field of the unique hypercoagulability and thrombo-inflammatory responses associated with COVID-19.
